# Network dynamics and therapeutic aspects of mRNA and protein markers with the recurrence sites of pancreatic cancer

**DOI:** 10.1016/j.heliyon.2024.e31437

**Published:** 2024-05-17

**Authors:** Animesh Acharjee, Daniella Okyere, Dipanwita Nath, Shruti Nagar, Georgios V. Gkoutos

**Affiliations:** a Institute of Cancer and Genomic Sciences, University of Birmingham, Birmingham, United Kingdom; b MRC Health Data Research UK (HDR UK), Birmingham, United Kingdom; c Institute of Translational Medicine, University Hospitals Birmingham NHS, Foundation Trust, B15 2TT, United Kingdom; d Centre for Health Data Research, University of Birmingham, B15 2TT, United Kingdom; e Eureka Tutorials, Muzaffarnagar, U.P., 251201, India

**Keywords:** PDAC, Canonical correlation analysis, Multi-omics, Translational research

## Abstract

Pancreatic ductal adenocarcinoma (PDAC) is a deadly disease that typically manifests late patient presentation and poor outcomes. Furthermore, PDAC recurrence is a common challenge. Distinct patterns of PDAC recurrence have been associated with differential activation of immune pathway-related genes and specific inflammatory responses in their tumour microenvironment. However, the molecular associations between and within cellular components that underpin PDAC recurrence require further development, especially from a multi-omics integration perspective. In this study, we identified stable molecular associations across multiple PDAC recurrences and utilised integrative analytics to identify stable and novel associations via simultaneous feature selection. Spatial transcriptome and proteome datasets were used to perform univariate analysis, Spearman partial correlation analysis, and univariate analyses by Machine Learning methods, including regularised canonical correlation analysis and sparse partial least squares. Furthermore, networks were constructed for reported and new stable associations. Our findings revealed gene and protein associations across multiple PDAC recurrence groups, which can provide a better understanding of the multi-layer disease mechanisms that contribute to PDAC recurrence. These findings may help to provide novel association targets for clinical studies for constructing precision medicine and personalised surveillance tools for patients with PDAC recurrence.

## List of abbreviations

APUD cells =: Amine precursor uptake and decarboxylation cells
B2M =: Beta-2-microglobulin factor 1 or SDF-1
CAF =: cancer-associated fibroblasts
CCL5 =: Chemokine (C–C motif) ligand 5
CD8 =: Cluster of differentiation 8 encodes transmembrane glycoprotein
CCA =: Canonical correlation analysis
CD66b =: Cluster of differentiation 66b
CD11c =: Cluster of differentiation 11c
CorALS =: Correlation analysis of large-scale systems
CV =: Cross validation
CXCL1 =: Chemokine (C-X-C motif) ligand 1 (also known as GRO-α)
CXCL2 =: Chemokine (C-X-C motif) ligand 2 (also known as GRO-β)
CXCL8 =: Chemokine (C-X-C motif) ligand 8 (also known as interleukin-8 or IL-8)
CXCL12 =: Chemokine (C-X-C motif) ligand 12 (also known as stromal cell-derived
D =: Discovery cohort
FAP-alpha =: Fibroblast activation protein alpha
FOXM1 =: Forkhead box protein M1
GTP =: Guanosine triphosphate
GDP =: Guanosine diphosphate
GEO =: Gene Expression Omnibus
GRU =: Gated recurrent unit
GZMB =: Granzyme B
HAV.CR2 =: Hepatitis A virus cellular receptor 2 protein coding gene
HLA.DQ =: Human leukocyte antigen DQ coding gene
IFN-γ =: Interferon gamma
ISG =: Interferon stimulated gene
ISG15 =: Interferon stimulated gene 15 protein
IQR =: Interquartile range
ITGAM =: Integrin subunit alpha M
JAK =: Janus kinase protein
LASSO =: Least absolute shrinkage and selection operator
LY6E =: Lymphocyte antigen 6 family member E
α[SMA] =: Alpha-smooth muscle actin
MHC-I =: Major histocompatibility complex class 1
MDSC =: Myeloid-derived suppressor cells
miRNA =: microRNA
NK cells =: Natural killer cells
PDAC =: Pancreatic ductal adenocarcinoma
PaCSCs =: Pancreatic cancer stem cells
PECAM =: Platelet endothelial cell adhesion molecule
PECAM1 =: Platelet endothelial cell adhesion molecule-1
RAS =: Rat sarcoma, a proto-oncogene
rCCA =: regularised canonical correlation analysis
rs =: Spearman correlation coefficient
SPLS =: Sparse partial least squares
S =: Shrinkage
SCFA =: Short chain fatty acid
STAT =: Signal transducer and activator of transcription gene
STING =: Stimulator of interferon genes
TCGA =: The Cancer Genome Atlas
TME =: Tumour microenvironment
TAM =: Tumour-associated macrophage
V1 =: Validation 1 cohort
V2 =: Validation 2 cohort
V3 =: Validation 3 cohort

## 1 Introduction

Pancreatic cancer is one of the most lethal tumours and the seventh leading cause of cancer-related mortality worldwide [1]. With an increasing incidence, global cancer deaths attributable to pancreatic cancer are expected to rise further by 2025 [2,3]. Despite improvements in surgical, adjuvant multiagent chemotherapy and diagnostic techniques [4], the 5-year survival rate for pancreatic cancer (13 %) remains the lowest of all cancer types and has stagnated over the last half century [5,6]. Approximately 80 % of pancreatic cancer patients are diagnosed with advanced disease, or distant metastases that confer poor patient prognosis with 5-year survival rate at 3 % [7,8]. Moreover, disease recurrence happens for approximately 90 % of pancreatic cancer patients within the first year of diagnosis [9,10]. Early diagnosis of pancreatic cancer in patients improves the curative effects of combinational surgery, radiotherapy and chemotherapy [11]. However, the high heterogeneity of pancreatic cancer, particularly pancreatic ductal adenocarcinoma (PDAC) as the most common type of pancreatic neoplasm [4], and poor patient outcomes emphasises the current challenges for early diagnosis and effective treatment. Thus, pancreatic cancer research is important for exploiting the potential of precision medicine and personalised surveillance strategies [12,13]. Especially, multiple biomarker and diagnostic tools can be used to detect non-invasive markers and achieve early stratification of pancreatic cancer patients [[14], [15], [16]].

To work towards the discovery of novel diagnostic and therapeutic targets for PDAC, multi-omics integration techniques can be utilised to elucidate a holistic view of the complex biological processes that contribute to PDAC disease mechanisms [17]. Developments in high-throughput sequencing technologies have contributed to the increased availability of omics data and have provided new opportunities for personalised medicine. In the literature omics integration and data fusion were used interchangeably and different strategies were developed, for example: early integration, intermediate integration, and late integration. More recently, intermediate integration methods have gained considerable interest due to their potential to assess the relationship between multi-omics data (inter-connectivity), while simultaneously revealing complementary information within each omics data set (intra-connectivity) without prior selection of outcome variables (features) [18]. Each omics data set represented information from a biological layer (for example, the transcriptome, proteome, metabolome, microbiome, lipidome). Intermediate integration methods include canonical correlation analysis (CCA) [19], whereby the correlation between linear combinations of features from each dataset is maximised, and partial least squares (PLS) [20], where covariance is maximised in this case. However, CCA implementation suffers in high-dimensional datasets where a greater number of features compared to samples available occurs (p ≫ n), and in datasets with high multicollinearity and noise [21]. Consequently, an extended version of traditional CCA with regularisation penalties, namely regularised CCA (rCCA), can be applied for effective multi-omics integration [22]. Conversely, traditional PLS can effectively integrate highly dimensional datasets but interpretability can be limited [21]. Sparse partial least squares (SPLS) [21,23] can therefore be applied to extend traditional PLS by including least absolute shrinkage and selection operator (LASSO) penalty [24]. Additionally, SPLS enables simultaneous feature selection in the separate datasets while regularised CCA does not [21]. Importantly, group feature connections can be explored using these methods unlike univariate correlation analysis techniques. Moreover, further experiments can be conducted to validate the discovered associations.

As previously indicated, increased availability multi-omics data sets are available, especially from online public repositories, such as, The Cancer Genome Atlas (TCGA; [25]) and the Gene Expression Omnibus (GEO; [26]). Yet, multi-omics integration can be complicated by noisy nature of biomedical data. As a result, the curse of dimensionality problem is presented, whereby a greater number of features compared to cases occurs [27]. Consequently, multicollinearity occurs in high dimensional data sets, thus filtering [28] of multi-omics data sets is required as a prerequisite for multi-omics integration strategies [29]. To achieve this important step, this is where ML and network-based methods are utilised to perform automatic feature selection whilst unveiling complex molecular mechanisms. For example, Placido et al., used longitudinal clinical data from Danish and American patients, including more than 25,000 pancreatic cancer cases, to perform patient risk prediction for pancreatic cancer and construct affordable surveillance regimens for early disease diagnosis [30]. This was performed by applying newly established methods using ML techniques, like gated recurrent unit (GRU) models [30]. On the other hand, Jagtap et al., used multi-omics network-based methodologies to perform microRNA (miRNA) and mRNA inference using TCGA pancreatic data sets [31]. Additionally, biological prior knowledge was included in the BRANET model, the performance of BRANET miRNA-mRNA regulatory network inference was assessed [32]. On the other hand, Becker et al., developed Correlation Analysis of Large-scale (biological) Systems (CorALS), a Python package that supported correlation analysis, feature selection using highly correlated variable (feature) pairs, and network construction using selected features from high-dimensional multi-omics pregnancy data sets [33]. Although complete correlation was used in this study, partial correlation can be utilised to distinguish between direct and indirect associations [34]. In turn, these studies have addressed the multicollinearity issue present when using biomedical data via feature extraction. ‬‬‬‬‬‬‬‬‬‬‬‬‬‬‬‬‬‬‬‬‬‬‬‬‬‬‬‬‬‬‬‬‬‬‬‬‬‬‬‬‬‬‬‬‬‬‬‬‬‬‬‬‬‬‬‬‬‬‬‬‬‬‬‬‬‬‬‬‬‬‬‬‬‬‬‬‬‬‬‬‬‬‬‬‬‬‬‬‬‬‬‬‬‬‬‬

Spatially organised multi-omics PDAC data sets with distinct recurrence patterns (including, liver, local, lung, peritoneal and no recurrence) were used in previous analyses and differential activation of immune pathway-related genes and unique stromal/inflammatory responses in the PDAC tumour microenvironment (TME) have were revealed as key players for distinct PDAC recurrence patterns [[35], [36], [37]]. However, the molecular mechanisms underpinning PDAC recurrence patterns are yet to be fully identified. Thus, we investigated associations present within and between gene expression and protein abundance data from Karamitopoulou et al., and publicly available datasets using concerted univariate and multivariate correlation analyses methods. In our study we have two objectives. Here, we first aimed to identify stable molecular associations across multiple recurrence sites in PDAC. This was performed by gene expression and protein abundance value transformation using Spearman partial correlation analysis. Consequently, networks were built to visualise stable univariate associations within and between multi-omics data sets. Second, we aimed to fuse or integrate two different multi-omics data (proteins vs. genes) using a ML integration framework. Multivariate canonical correlation techniques, including rCCA and SPLS, were implemented to identify group feature associations with simultaneous feature selection using SPLS. As a result, the stable associations identified in objective 1 (phase 1) were validated and complemented by these multivariate approaches. Moreover, datasets from TCGA and GEO repositories were used to further validate our findings (same as objective 1).

## 2 Results

### 2.1 Clinical data analysis

Each PDAC patient was classified as High-, Intermediate-, and Low-grade based on previously reported criteria and patient categories dependent upon the number of tumour buds identified [38,39]. A comparison of pathological and clinical features between High-, Intermediate-, and Low-grade patient groups for the Discovery cohort is presented in Table 1. No significant relationship was observed between tumour size differentiation, gender and organ recurrence site pattern for the Discovery and Validation 1 cohorts (Supplementary Table 4). However, High-grade patients had lower overall survival (OS) compared to Intermediate- and Low-grade patients for both Discovery and Validation 1 cohorts. Similarly, this trend was shown for disease-free survival (DFS) for Discovery and Validation 1 cohorts. Similar ages between the High-, Intermediate-, and Low-grade cases were observed for Discovery and Validation 1 cohorts. Yet in the Discovery cohort, the High-grade group constituted younger patients compared to both Intermediate- and Low-grade groups, whereas younger patients formed the Low-grade group compared to Intermediate- and High-grade groups in Validation 1 cohort. In the Validation 2 cohort [40], statistically significant differences between the Germany (n = 45), Maryland (n = 27), and combined (n = 72) cohorts were observed for the age variable only and were comparable with patient ages in the Discovery and Validation 1 cohorts. Likewise, Validation 3 cohort [41] patients had similar ages to Discovery, Validation 1 and Validation 2 cohort patients. On the other hand, compared to Low-grade cases, the High-grade group displayed considerable significantly increased CA19-9 values, whilst the Intermediate-grade cases had significantly reduced CA19-9 values compared to the High- and Low-grade groups for both Discovery and Validation 1 cohorts. Both buds number ×20 and budding ITBCC were significantly increased in the High-grade group compared to the Intermediate- and Low-grade cases for both the Discovery and Validation 1 cohorts. Two patients from the Validation 3 cohort had missing gene and protein expression data and were excluded from analyses [41].

**Table 1:** Comparison of clinical and pathological variables between High-, Intermediate-, and Low-grade budding patients for Discovery dataset (n = 284). Quantitative data are presented as group medians and interquartile range (IQR) values are specified in brackets.

Characteristic	High grade	Intermediate grade	Low grade	p-value
Number (%)	101 (35.6)	116 (40.8)	67 (23.6)	
OS (months)	17 (8–28)	19 (15–28)	19 (12.5–63.5)	<2.2e-16
DFS (months)	12 (4–19)	13.5 (7–20)	14 (7–60)	<2.2e-16
Sex, n (%)				0.352
F	43 (15.1)	61 (21.5)	36 (12.7)	
M	57 (20.1)	55 (19.4)	31 (10.9)	
Missing	1 (0.3)	0	0	
Age	65 (58–74)	67 (64–72)	66 (58–76)	<2.2e-16
CA19-9 (U/mL)	564 (137.8–1500)	151 (100–371.8)	200 (137–853.8)	<2.2e-16
Size (mm)	30 (25–45)	30 (30–40)	30 (25–35)	<2.2e-16
Buds Number x20	18 (8–35)	11 (5.75–19)	8 (2–19)	<2.2e-16
Budding ITBCC	18.2 (6.6–28.9)	9.1 (4.98–16.7)	6.6 (1.7–15.7)	<2.2e-16
Budding category, n (%)				0.001
1	18 (17.8)	28 (24.1)	27 (40.3)	
2	20 (19.8)	37 (31.9)	18 (26.9)	
3	63 (62.4)	51 (44.0)	22 (32.8)	
Organ, n (%)				0.109
Liver	33 (32.7)	44 (37.9)	16 (23.9)	
Local	10 (9.9)	12 (10.3)	9 (13.4)	
Lung	15 (14.9)	24 (20.7)	10 (14.9)	
Other	20 (19.8)	10 (8.6)	8 (11.9)	
No recurrence	23 (22.8)	26 (22.4)	24 (35.8)	

Abbreviations: OS, overall survival; DFS, disease free survival.

### 2.2 Global gene expression patterns

A heat map was used to explore global gene expression and clustering patterns, and mean expression for the genes included in the Discovery and Validation 1 data sets (Fig. 3). The liver recurrence site for both the Discovery and Validation 1 data sets exhibited high abundance of downregulated gene expression, whereas the no recurrence site revealed significant upregulated genes (Fig. 3). The B2M gene had highest mean expression in both Discovery and Validation 1 sets. Hierarchical clustering of the genes showed two main clusters of genes for both the Discovery and Validation 1 data sets (Fig. 3).

### 2.3 Partial correlation analysis identified stable gene and protein interactions

To investigate stable associations or consistent connections within (intra-connectivity) and between (inter-connectivity) transcriptome and proteome data sets, we computed Spearman partial correlations across the different recurrence sites for the Discovery, Validation 1, Validation 2, and Validation 3 data sets (Table 2). We considered a Spearman partial correlation coefficient threshold >0.6. All mRNA, protein and combined association correlations for Discovery, Validation 1, Validation 2 and validation 3 data sets are outlined in supplemental tables 1.1, 1.2 and 1.3, respectively. We focused on reported genes and proteins identified in Ref. [35]. In the Discovery data set, the FAP-alpha – SMA protein connection occurred in both PDACs with lung and no recurrences. This protein connection was also identified in the Validation 2 data set for PDACs with no recurrence. For the Validation 1 data set along with the Validation 3 data set, the FAP-alpha – SMA protein connection was found in PDACs with local recurrences. For both the Discovery and Validation 1 data sets, ITGAM – PECAM1 gene association was consistent across PDACs with liver and local recurrences. Additionally, this gene association occurred in PDACs with peritoneal and no recurrences for the Validation 1 data set (Table 2). Between the Discovery and Validation 1 proteome and transcriptome data sets, the CD11c - FAP-alpha and SMA – FAP-alpha mRNA-protein connections occurred in PDACs with local recurrences. One negative correlation score was found for Validation 1 cohort peritoneal recurrence CD11c - FAP-alpha connection (Table 2). The CCL5 – CXCL10 mRNA-protein connection was identified between the Discovery and Validation 1 proteome and transcriptome data sets (Table 2). The gene-gene and protein-protein marker associations network for the Validation 1 dataset are shown in Fig. 4.

**Table 2:** Consistent Spearman partial correlation connections in all data sets.

Marker type	Markers	Recurrence site	Discovery (r_s_)	Validation 1 (r_s_)	GEO (r_s_)	TCGA (r_s_)
Protein	FAP-alpha – SMA	Lung	0.71			
		Local		0.61		0.70
		No recurrence	0.70		0.62	
mRNA	ITGAM – PECAM1	Liver	0.60	0.71		
		Local	0.66	0.70		
		Peritoneal/other		0.71		
		No recurrence		0.63		
Combined	CD11c - FAP-alpha	Local	0.70	−0.66		
	SMA – FAP-alpha	Local	0.69	0.80		
	CCL5 – CXCL10	Peritoneal	0.62	0.87		

Abbreviations: r_s_; Spearman correlation coefficient, SMA; αSMA.

### 2.4 Multivariate canonical correlation analysis identified novel associations identified using all genes and proteins

To explore novel associations across the different recurrence sites between the Discovery, Validation 1, Validation 2, and Validation 3 data sets, rCCA and SPLS methods were used (Table 3). Novel associations with correlation threshold >0.6 were included and information about all genes and proteins included in analyses are outlined in Supplemental Tables 2 and 3, respectively. Using rCCA, STAT1 – CCL5 novel association was discovered across PDACs with local and no recurrences for Validation 2 and Validation 3 data sets. Moreover, HAVCR2 – PECAM1 novel association was identified across PDACs with local and no recurrences for Validation 1 and Validation 2 data sets (Table 3). SPLS analysis revealed CD8A – ITGAM novel connection in PDACs with liver recurrence for the Discovery set, and STAT2 – PECAM1 novel connection across PDACs with liver and peritoneal recurrences for the Discovery and Validation 1 data sets (Table 3). For the novel associations, ITGAM and PECAM1 genes had the highest node degree value of three. HAVCR2, STAT2, CD8A and STAT1 had the lowest node degree value of one. Overall summary for the associated stable markers identified from all methods employed in this study is depicted in Fig. 4 4(A-D). Also, common markers are shown in Fig. 4 4E as a Venn diagram.

**Table 3:** Overall summary of novel connections identified for mRNA markers only across multiple recurrence sites using regularised canonical correlation analysis (rCCA) and sparse partial least squares (SPLS).

Analysis performed	Threshold	Recurrence site	Data set	Gene	Novel association with reported genes in *Karamitopoulou* et al.*,* (2023)
rCCA	0.6	Local	TCGA	STAT1	CCL5
	0.7	No recurrence	GEO		
	0.6	Liver	Validation 1	HAVCR2	PECAM1
	0.7	No recurrence	GEO		
SPLS	0.7	Liver	Discovery	CD8A	ITGAM
	0.7	Liver	Discovery	STAT2	PECAM1
	0.6	Peritoneal	Validation 1		

### 2.5 Novel associations gene expression distribution

To assess the contribution of genes identified we analysed weights (or coefficients) from rCCA and SPLS gene loading weight results. In the Discovery data cohort for PDACs with liver recurrences, STAT2 had the highest weight contribution (weight = 0.22) compared to other genes included in the novel connections identified (Fig. 4F). In the same dataset and recurrence site, CD8A was identified as part of a novel connection and had high weight contribution (weight = 0.15). The STAT1 gene in the TCGA (Validation 3) data cohort for PDACs with local recurrences had the lowest and a negative weight contribution (weight = −0.001), and in the GEO (Validation 2) data cohort with the weight contribution for this gene (weight = −0.021) followed a similar pattern. Statistically significant differences between the distinct recurrence groups were assessed for the genes involved in the novel associations. Mean expression of STAT2 was upregulated in PDACs with peritoneal compared to liver recurrences. PDACs with no recurrence showed downregulated STAT1 and HAVCR2 expression when compared to PDACs with local and liver recurrences.

### 2.6 Biological interpretation with the markers

In this study we have analysed data of postoperative PDAC symptoms, recurrence and correlation among various genes and proteins across different patient cohorts.

Fig. 5 explains how each of the biomolecules are capable of triggering PDAC recurrence through various proteogenomic pathways. FOXM1 is an important protein in this parameter as mentioned by Liu et al., [42]. FOXM1 expression negatively impacts the level of phosphorylated signal transducer and activator of transcription 1 (pSTAT1) in human pancreatic cancer tissues. The principal function of JAK/STAT pathway which is to regulate and control processes such as stem cell maintenance, hematopoiesis and the inflammatory responses gets affected henceforth. The studies conducted by Bektas et al., indicates that chemical inhibitors of FOXM1 can be used in anticancer therapeutics [43]. Next, we have the STAT1-CCL5 axis which is a very important modulator in the recurrence of PDAC via the lymphatic system metastasising into peritoneal tumours, including colorectal cancer. Hence STAT1-CCL5 levels can act as valuable biomarkers for postoperative recurrent PDAC screening as reported by Niu et al., [44]. STAT1 gene can enhance immune surveillance by increasing the expression of several interferon stimulating genes and can increase the tumour-killing potential of the natural killer (NK) cells. As detailed in the study conducted by Romero et al., expression of CCL5 along with chemokine CCL4, CCL9 and CCL10 is strongly associated with CD8^+^ T cell infiltration in resectable and metastatic PDAC tumours with active anti-tumour phenotypes [45]. Plasma levels of interleukins and chemokines have been reported by Ponziani et al., to be higher in hepatocellular carcinoma [46].

The oral and intestinal micro-ecology also have a significant role to play in the PDAC tumour microenvironment. *Helicobacter pylori* is an important factor for the proliferation of peptic ulcers and gastric cancers and has strong correlation with the urea cycle. A urea screening test is used as a screening technique for the presence of *H. pylori* [47]. Its association with PDAC is through the HLA.DQ and CCL5 biomolecules. *Enterococcus faecalis* have been reported by Wang et al., to have infected macrophages with the expression of LY6E gene during peritoneal metastasis [48]. Short chain fatty acids like Butyrate are produced in the body when gut microbiome like *Bifidobacterium* helps breakdown dietary fibers in the colon. 11 strains of *Bifidobacterium* have been reported by Yang et al., that has the potential to increase the levels of CD8^+^ T cells [49]. Kang et al., detailed in their study that if colorectal cancer cells are exposed to butyrate, it can develop a certain amount of butyrate resistance in the body. This can increase the tumour necrosis factor in the body, resulting in tumour progression and apoptosis.

CD11c, a marker of dendritic cells, interacting with GZMB, a marker of cytotoxic T cells, highlights potential interactions between the immune microenvironment and tumour cells. Modulating this interaction could enhance anti-tumour immune responses and overcome immune evasion mechanisms employed by PDAC cells, leading to improved response to immunotherapy and immune checkpoint inhibitors. CD66b is associated with neutrophils, while CD11c is expressed on dendritic cells [50]. The interaction between CD66b and CD11c mRNA suggests potential crosstalk between neutrophils and dendritic cells within the tumour microenvironment. Targeting this interaction could modulate the inflammatory response and enhance antigen presentation, leading to improved immune surveillance and anti-tumour immunity.

The interaction between smooth muscle actin (SMA) and fibroblast activation protein-alpha (FAP-alpha) mRNA holds significant implications, which play a crucial role in tumour stroma remodelling. This interaction can promote angiogenesis [51].Thus, targeting this interaction can inhibit tumour progression and enhance the efficacy of other therapeutic modalities, such as chemotherapy, radiation therapy, and immunotherapy. Evaluating the expression levels of SMA and FAP-alpha in tumour biopsies or circulating biomarkers may help stratify patients based on their risk profile and hence help take informed decisions.

CD11c-expressing dendritic cells play a pivotal role in immune surveillance and antigen presentation within the tumour microenvironment. Conversely, FAP-alpha is predominantly expressed on CAFs (cancer-associated fibroblasts), which contribute to tumour growth, metastasis, and immunosuppression [52]. The interaction between CD11c and FAP-alpha mRNA suggests a potential crosstalk between dendritic cells and CAFs, shaping the immunosuppressive microenvironment in PDAC. Clinically, this interaction may contribute to immune evasion and hinder the efficacy of immunotherapies, highlighting the importance of understanding and targeting the tumour stroma for improving treatment outcomes.

ITGAM which encodes for CD11b, is a cell surface receptor primarily expressed on leukocytes, including monocytes, macrophages, and neutrophils. PECAM1, on the other hand, is a cell adhesion molecule expressed on endothelial cells and leukocytes. The interaction between ITGAM and PECAM1 facilitates tumour cell adhesion to endothelial cells and promotes tumour cell invasion through the endothelial barrier. Clinically, this interaction may contribute to the dissemination of cancer cells from the primary tumour site to distant organs, leading to metastasis and recurrence.

## 3 Discussion

Pancreatic cancer is a lethal disease that is typically diagnosed at advanced stages with poor patient outcomes. Moreover, frequent disease recurrence occurs for patients with pancreatic cancer. As a result, there is a need for effective diagnostic and therapeutic tools for improved patient prognosis, especially by leveraging precision medicine and personalised disease management methods. However, a developed understanding of biological relationships within and between different biological layers responsible for PDAC disease recurrence mechanisms is required. To this end, we aimed to identify and visualise consistent molecule connections across multiple PDAC recurrences, including, liver, lung, local, peritoneal, and no recurrence, by using univariate and multivariate correlation analysis workflows and network construction. To do so, Spearman partial correlation analysis was employed to identify stable associated gene and protein markers that were consistent across distinct PDAC recurrences using spatial transcriptome and proteome data sets. Then, Cytoscape was used to construct stable networks. Moreover, we purposed to identify novel associations, as well as groups of stable connections, across multiple PDAC recurrences using ML-based intermediate integration techniques. To achieve this, multivariate canonical correlation analysis methods, including rCCA and SPLS were used. To validate these findings, publicly available data sets from TCGA and GEO repositories were acquired and utilised. Therefore, across the distinct PDAC recurrences, stable protein, mRNA, and protein-mRNA (combined) markers were discovered. Additionally, novel gene associations including, STAT1, HAVCR2, CD8A, and STAT2 genes, were discovered using rCCA and SPLS.

Stable associations, including inter- and intra-connections between proteome and transcriptome data, across distinct PDAC recurrences were identified. While multi-layer associations were also identified using pancreatic cancer TCGA datasets by Jagtap et al.*,* the associations revealed in this study did not overlap with those found in this study [32]. The observed difference in associations identified could be attributable to the use of mRNA and protein data sets in this study, compared to the use of miRNA and mRNA data sets to perform network-based multi-omics integration. Alternatively, the difference could be due to a Spearman partial correlation threshold being used in this study compared to the use of a Pearson correlation threshold and biological *a priori* knowledge for important association determination and network construction [32]. Consequently, the strength of monotonic associations between features were measured using Spearman correlation as compared with linear associations using Pearson correlation [53]. These results suggest the importance of investigating the multi-layer associations that exist within and between different layers to develop a more comprehensive picture of relationships that could be responsible for pancreatic cancer pathogenesis. However, further statistical analyses would be required to determine the importance of associations between miRNA-MRNA and mRNA-protein associations. ‬‬‬‬‬‬‬‬‬‬‬‬‬‬‬‬‬‬‬‬‬‬‬‬‬‬‬‬‬‬‬‬‬‬‬‬‬‬‬‬‬‬‬‬‬‬‬‬‬‬‬‬‬‬‬‬‬‬‬‬‬‬‬‬‬‬‬‬‬‬‬‬‬‬‬‬‬‬‬‬‬‬‬‬‬‬‬‬‬‬‬‬‬‬‬‬‬‬‬‬‬‬‬‬‬‬‬‬‬‬‬‬‬‬‬‬‬‬‬‬‬‬‬‬‬‬‬‬‬‬‬‬‬‬‬‬‬‬‬‬‬‬‬‬‬‬‬‬‬‬‬‬‬‬‬‬‬‬‬‬‬‬‬‬‬‬‬‬‬‬‬‬‬‬‬‬‬‬‬‬‬‬‬‬‬‬‬‬‬‬‬‬‬‬‬‬‬‬‬‬‬‬‬‬‬‬‬‬‬‬‬‬‬‬‬‬

Novel associations across multiple distinct PDAC recurrences were discovered using different mRNA data cohorts. The STAT1-CCL5 association identified in PDACs with local recurrences support previous findings by Niu et al., although this connection was observed in the context of colorectal cancer (10.13039/100012928CRC) [44]. Both STAT1 and CCL5 are involved in cytokine signalling and coding for chemokines. Upregulation of this biomarker association axis in CRC was observed to promote colon cancer cell proliferation [44]. Thus, activity of the STAT1-CCL5 connection shown in CRC could help to explain disease mechanisms that contribute to PDAC local recurrence patterns via probable enhanced proliferation of pancreatic cancer cells. These results imply that associations identified in this study are also related to the disease biology of other gastrointestinal cancers. This could provide evidence for evaluation of the STAT1-CCL5 association in upcoming clinical studies for investigating the development of novel therapeutic tools that target this connection. On the other hand, unphosphorylated STAT1 can promote forkhead box protein M1 (FOXM1) upregulation, which can lead to enhanced patient chemoresistance to gemcitabine drug in pancreatic cancer [42]. However, interferon γ (IFNγ) has been observed to promote STAT1 phosphorylation, which in turn can lead to FOXM1 downregulation in pancreatic cancer patients [42,54]. These results suggest the potential benefit of IFNγ for PDAC patients with local recurrences. However, further molecular studies are required to assess the therapeutic benefit that could be provided by IFNγ.

For the baseline characteristics, CA19-9 was observed to be lower for the Intermediate-grade group compared to Low-grade cases. These results differ from those obtained by Dong et al., while High-grade pancreatic cancer cases were observed to have elevated levels of CA19-9 compared to Low-grade cases, the median CA19-9 levels increased with the tumour grade [55]. This unusual result could be caused by more Intermediate-grade PDAC (n = 116) cases compared to the Low-grade PDAC cases (n = 67) in this study. These results suggest that CA19-9 levels can display wide variability that can impact median values depending on sample sizes.

Correlation analysis was performed to identify univariate and multivariate correlations within and between protein and mRNA data sets. Although correlation analysis can be used to better our understanding of the relationships that occur between gene and protein markers, correlation does not mean causation [56]. Therefore, careful interpretation and understanding of these findings are required in the context of pancreatic cancer recurrence patterns and therapeutic aspects. These results indicate that further study is required to strengthen evidence for the associations identified and the potential for clinical translation in practice. However, the provision of open-source scripts and data sets used in our analyses could positively contribute to Findability, Accessibility, Interoperability, and Reusability (FAIR) guiding principles in scientific data management [57]. This in turn could improve the clinical translation of the findings reported in this study, yet future investigations are still required.

Alongside correlation analysis, feature association selection was performed using top correlated features and ML techniques. To assess the stability of feature selection, further tests are required to benchmark the techniques used for selection [58]. The biological relevance of feature selection stability could be associated with prediction model construction for potential beneficial use in clinical practice. Novel gene associations across the PDAC recurrences were identified. However, novel protein associations were not revealed because we proteome data for the Validation 2 and Validation 3 data sets related to PDAC were not accessible. However, novel protein associations can be explored in future studies with growing availability of publicly available multi-omics data sets.

Protein and gene associations have been identified for different PDAC recurrence types. However, another biological layer, for example, the microbiome, can be incorporated in future multi-omics integration studies to facilitate exploration of different types of associations that could contribute to PDAC disease recurrence patterns. By doing so, such future studies could aid to further increase our understanding of the disease pathways that potentially contribute to PDAC recurrence patterns. In turn, these multi-omics integration studies could provide evidence for other novel association features that could be candidates for precision medicine therapy strategies for pancreatic cancer patients. Furthermore, future multi-omics studies with the use of longitudinal protein and gene data sets could help to provide a more comprehensive view of the associations within and between distinct molecular layers [59]. Consequently, further information on the dynamics of networks could be investigated in future studies.

Limitations of this study include a lack of data comparability between the Discovery and Validation 1, 2 and 3 data sets. Although both the Discovery and Validation 1 data sets include clinical, spatial transcriptome and proteome data for liver-, local-, lung-, peritoneal, and no-recurrence PDACs, Validation 2 and 3 data sets were limited to no-recurrence and local-recurrence data, respectively. Data from large clinical trials would be required, yet the 5-year survival rate for pancreatic cancer is the lowest of all cancers [5] and consequently the results are limited by the number of patient samples.

Strengths of this study include the application of multivariate modelling together with univariate correlation methods. Even though our data was limited, we attempted to discover novel gene-gene, protein-protein, and gene-protein associations that could be leads for future study, as well as the stability of these leads across multiple different cohort groups. Those stable leads will also provide good candidates for translational research and the development of personalised surveillance tools for patients with PDAC recurrence.

### 3.1 Conclusion

Stable associations have been identified that were consistent across distinct patterns of PDAC recurrence. Moreover, novel associations that were identified across multiple PDAC recurrences were revealed. These findings may help to suggest candidate associations in further molecular investigations for personalised therapeutic options for PDAC patients, particularly in the context of specific recurrence patterns. However, further studies are required to investigate causal links from correlated associations.

## 4 Materials and methods

### 4.1 Public data sets

**Fig. 1:**
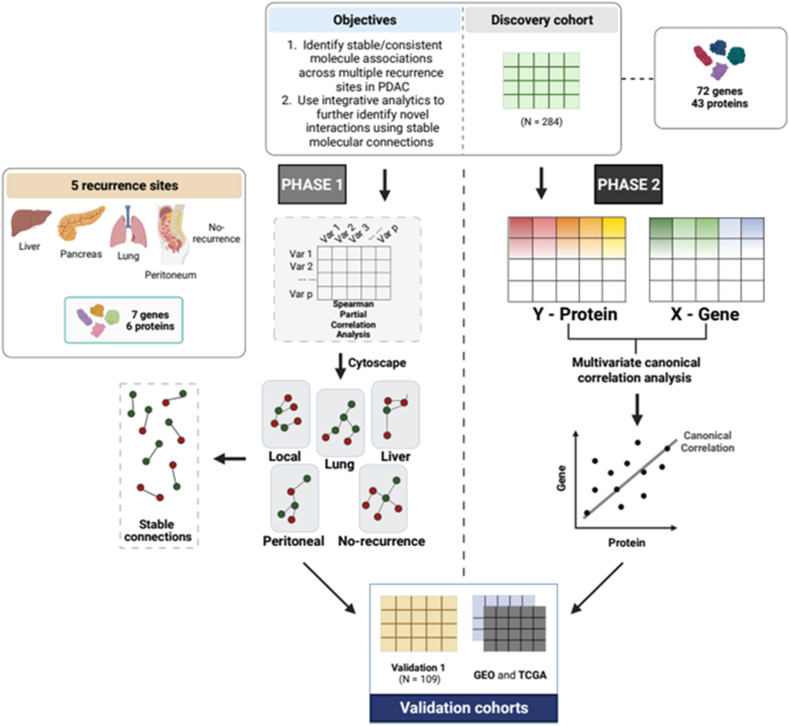
Design of study is shown. Spatial transcriptomic and proteomic data sets comprised of data from liver, local lung, peritoneal, and no recurrence sites, and were included in the Discovery and Validation 1 sets. Transcriptomic and proteomic data were included in the GEO and TCGA data sets. In phase 1, Spearman partial correlation analysis was conducted to find stable connections (Spearman's coefficient >0.7) using gene expression and protein abundance data both separately and combined. Subsequent network construction was performed using Cytoscape to visualise the stable associations. Similar correlation analysis and network construction were performed using validation data sets. In phase 2, multivariate canonical correlation analyses, including regularised canonical correlation analysis (CCA) and sparse partial least squares (PLS), were performed using the protein abundance and gene expression datasets as the Y and X matrices, respectively. The canonical correlations and loading vectors produced by regularised CCA and sparse PLS, respectively, were assessed to identify stable individual and grouped connections between the multi-omics datasets. These connections were validated using the same methods and validation data sets.

We have used publicly available data sets to perform the analysis. Gene expression, protein abundance, and clinical data constituted the cohorts we considered as Discovery and Validation 1, and were previously published [35]. The gene expression matrices employed in this study were obtained from GEO (https://www.ncbi.nlm.nih.gov/gds/?term=) and TCGA (https://www.cancer.gov/ccg/research/genome-sequencing/tcga) database repositories, and accessed on May 12, 2023 and May 29, 2023, respectively. Moreover, these datasets will be considered as Validation 2 and Validation 3 cohorts, respectively. Further information about all datasets used is summarised in Table 4. The *GEOquery* [60] and *TCGAbiolinks* [61] R Bioconductor tools were used to download and prepare relevant GEO and TCGA data, respectively. These datasets were used to investigate the associations between multiple markers and validate them. The study design is outlined in Fig. 1.

**Table 4:** Detailed information on the publicly available data sets including cohort description, data sources and recurrence sites are listed below.

Cohort description	Data source	Recurrence site used	References
Discovery cohort (N = 284)	*Karamitopoulou* et al.*; 2023*	Liver (N = 93)	[35]
		Lung (N = 49)	
		Local (N = 31)	
		Peritoneal/other (N = 38)	
		No-recurrence (N = 73)	
Validation cohort −1 (N = 109)	*Karamitopoulou* et al.*; 2023*	Liver (N = 33)	[35]
		Lung (N = 20)	
		Local (N = 14)	
		Peritoneal/other (N = 15)	
		No-recurrence (N = 27)	
Validation cohort −2 (N = 45)	GSE28735	No-recurrence (N = 45)	[40]
Validation cohort −3 (N = 183)	TCGA	Local (N = 183)	[62]

### 4.2 Data pre-processing

There were some missing values in the protein abundance Validation 1 cohort dataset and we used median imputation to estimate the missing value [63]. The percentage of the missing values was 0.9 and only 1 sample was imputed. This process was used for imputing the missing value in the IDO1 protein feature column based on the distribution of protein abundance values.

### 4.3 Statistical analysis

#### 4.3.1 Spearman partial correlation analysis

We used Spearman's rank correlation to explore associations between genes and proteins [35]. Reported genes (HLA-DQ, ITGAM, LY6E, CCL5, CD44, CXCL10 and PECAM1) and proteins (STING, CD66b, CD11c, α[SMA], FAP-alpha AND GZMB) with the highest discriminative ability across the PDAC recurrence groups from Ref. [35] were included. Spearman correlation does not rely on normality and linearity of associations. This process was performed to identify strongly correlated genes and proteins. Spearman partial correlation coefficients were calculated using the *ppcor* R package [64]. Normal Spearman correlation coefficients were calculated using the basic correlation function in R.

#### 4.3.2 Partial correlation based derived networks

A network [43] consists of vertices (nodes) and connections or interactions (edges). In our study, each of the nodes represents either mRNA or proteins. All possible permutations of mRNA and protein features were measured for their degree of relationship using partial Spearman correlation coefficients as calculated by using the ppcor R package [S. Kim (2015)]. In order to establish links between the features, multiple associations were considered. We used a significance threshold (alpha = 0.05) and only significant relationships (*p* < 0.05) were drawn. Cytoscape version 3.9.1 was used to create network visualisations, including the features as nodes and significant relationships, and analyse networks. To construct the correlation networks, network tables, including information about the source node, target node and partial correlation coefficient, were uploaded to Cytoscape. The default networks settings for the correlation networks created were adjusted for node characteristics, such as colour, size, and shape, and edge characteristics, including line type and thickness. Once completed, the correlation networks were exported to image from Cytoscape.

### 4.4 Overview of multi-omics integrative machine learning framework

**Fig. 2:**
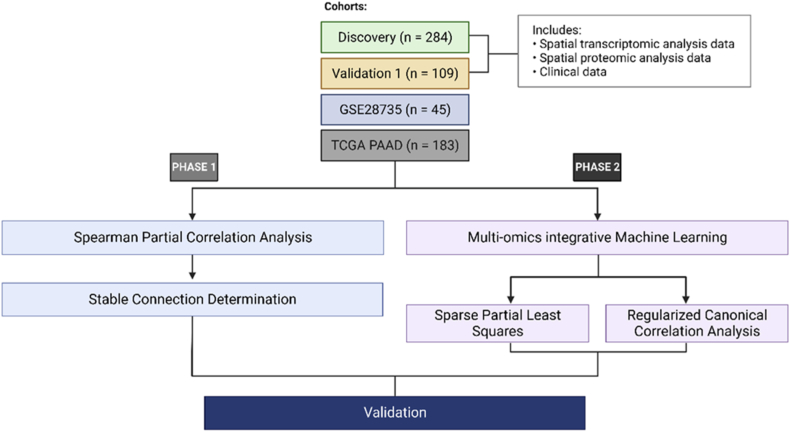
Outline of study workflow to discover and validate feature connections from multi-omics data sets including information for different recurrence sites.

A machine learning [[65], [66], [67]] framework was investigated for integrating high-dimensional multi-omics datasets, including gene expression and protein abundance. Our integration method comprises two parts: (1) regularised canonical correlation analysis (rCCA) [68] for determining groups of genomic variables (features) that associate with groups of proteomic features, and (2) sparse partial least squares (SPLS) [23] to simultaneously perform feature selection on both transcriptomic and proteomic data sets via least absolute shrinkage and selection operator (LASSO) penalisation [24], as well as identifying the relationship between the datasets. These methods were performed using the data sets for the liver, local, lung, peritoneal/other, and no recurrence sites to facilitate discovery to target approach (Fig. 2, phase 2). These results were then compared with that from the target to discovery approach (phase 1). We outlined both approaches in detail below and presented in Fig. 2.

**Fig. 3:**
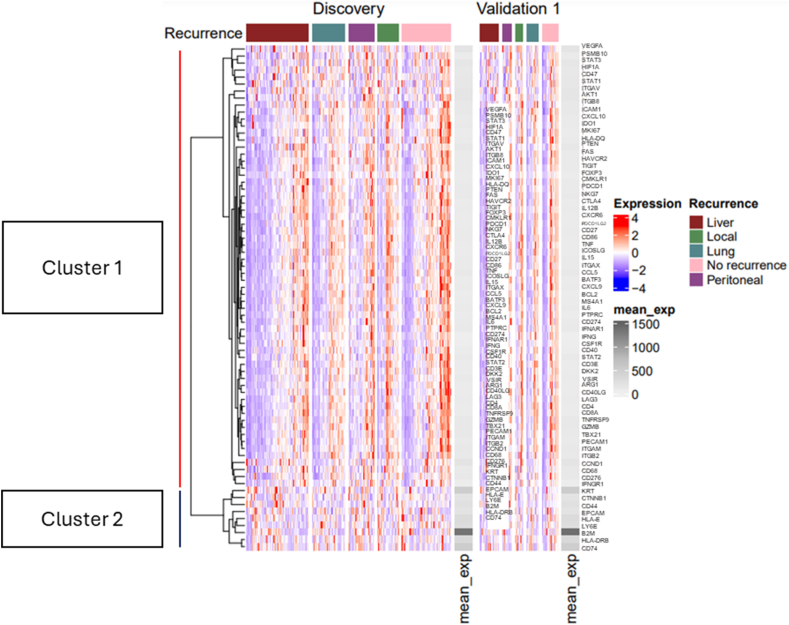
Heatmap and hierarchical clustering for Discovery and Validation 1 gene expression data sets. Gene expression values correspond to values of the heatmap and were obtained by scaling each gene row expression values. The clustering shows the closely related genes and the groups of closely related genes. Spearman distance with Wald D2 method was used for hierarchical clustering. Mean expression (*mean_exp*) of each gene is shown in grey side annotations for each data set. The colour gradient from blue to red shows low to high relative expression, and white indicates no expression. The sample columns are split according to the recurrence site for both data sets. The liver, local, lung, no recurrence, and peritoneal sites are coloured as red, green, blue, pink, and purple bars, respectively. Clusters 1 and 2 are shown within the legend left side. (For interpretation of the references to colour in this figure legend, the reader is referred to the Web version of this article.)

**Fig. 4:**
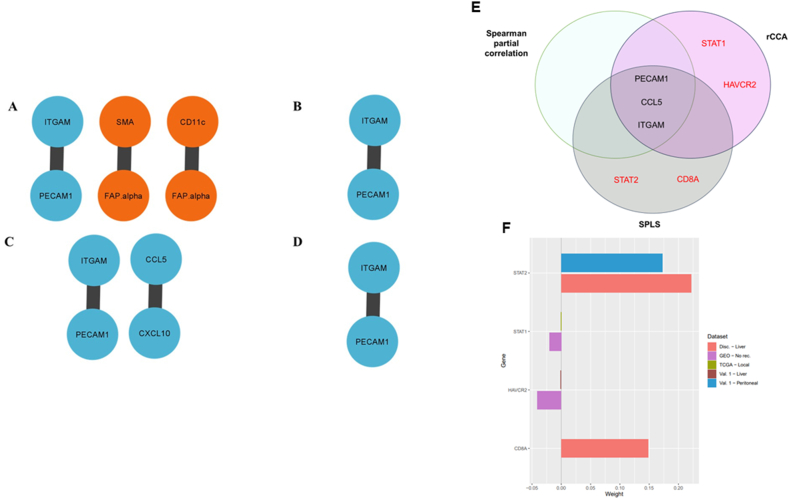
Gene and protein marker association network graphs showing (A) protein and gene connections for PDACs with local recurrences, (B) gene connections for PDACs with no recurrence, (C) gene connections for PDACs with peritoneal recurrences, and (D) gene connections for PDACs with liver recurrences from the Validation 1 cohort dataset. In (D) the dashed line represents a negatively correlated association. (E) Venn diagram showing shared genes between Spearman partial correlation analysis, regularised canonical correlation analysis (rCCA) and sparse partial least squares (SPLS). Novel associated genes are coloured in red. (F) Loading plot showing weight of genes from discovered connections for mRNA markers only across multiple recurrence sites using regularised canonical correlation analysis (rCCA) and sparse partial least squares (SPLS). (For interpretation of the references to colour in this figure legend, the reader is referred to the Web version of this article.)

**Fig. 5:**
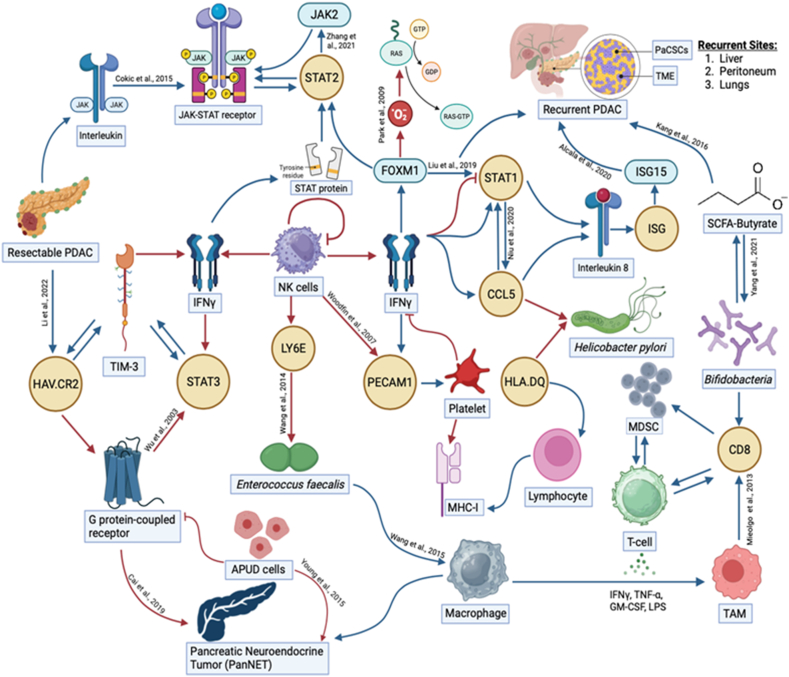
The proteogenomic insights on the biological interpretations of correlation of biomolecules in postoperative PDAC recurrence. PDAC =Pancreatic ductal adenocarcinoma, JAK = Janus kinase protein, STAT = Signal Transducer and Activator of Transcription gene, IFN-γ = Interferon gamma, NK cells = Natural Killer cells, RAS = Rat sarcoma, a proto-oncogene, GTP = Guanosine triphosphate, GDP = Guanosine diphosphate, FOXM1 = Forkhead box protein M1, PaCSCs = Pancreatic cancer stem cells, TME = Tumor microenvironment, ISG = Interferon stimulated gene, ISG15 = Interferon stimulated gene 15 protein, SCFA = Short chain fatty acid, CCL5 = Chemokine (C–C motif) ligand 5, CD8 = Cluster of Differentiation 8 encodes transmembrane glycoprotein, HAV.CR2 = Hepatitis A Virus Cellular Receptor 2 protein coding gene, HLA.DQ = Human Leukocyte Antigen DQ coding gene, MHC-I = Major histocompatibility complex class 1, MDSC = Myeloid-derived suppressor cells, TAM = Tumor-associated macrophage, LY6E = Lymphocyte Antigen 6 Family Member E, PECAM1 = Platelet endothelial cell adhesion molecule-1, APUD cells = Amine precursor uptake and decarboxylation cells.

#### 4.4.1 Regularised CCA

Regularised CCA [22] was used to determine multivariate-level correlations between gene expression and protein abundance data sets for the Discovery, and Validation 1, 2, and 3 cohorts. Classical CCA [19] maximises correlation between the two datasets by identifying linear projection of two sets of features into a shared transformed space [69]. Regularised CCA extends CCA by using ridge regression or L2 penalty for application to high-dimensional datasets [22]. The ridge regression method adds a multiple of the resulting identity matrix to perform penalisation of the correlation matrices. Alternatively, let us examine

$$A_{XX}\left(\lambda_{1}\right)=A_{XX\;}+\lambda_{1}I_{p}$$

and

$$A_{YY}\left(\lambda_{2}\right)=A_{YY}+\lambda_{2}I_{q}$$

where.

$A_{XX}$ and $A_{YY}$ denotes the sample correlation matrices for variable sets for X, of order n×p, and Y, of order n×q, respectively

$I_{p}$ and $I_{q}$ denotes the identity matrices of order p and q, respectively

$\lambda_{1}$ and $\lambda_{2}$ can take non-negative values

$A_{XX}\left(\lambda_{1}\right)$ and $A_{YY}\left(\lambda_{2}\right)$ denote regularised matrices [70].

To perform rCCA using the cross validation (Ridge) method, the hyperparameters were tuned using grid search method to determine the sparsity penalty parameters for gene expression ($\lambda_{1}$) and protein abundance ($\lambda_{2}$) and optimised using leave-one-out (LOO) internal cross validation method from *mixOmics* package [71]. Thus, using this method, we identified $\lambda_{1}$ and $\lambda_{2}$ hyperparameters using Ridge and shrinkage methods and a summary of the hyperparameter values used in this study can be found in Table 5. The canonical loadings, or correlations, were analysed for both Ridge and shrinkage penalisation methods applied to the multi-omics datasets using the *mixOmics* R package [71]. The upper and lower bounds for canonical loadings produced by Ridge and shrinkage methods for all proteome and transcriptome datasets are summarised in Table 6.

**Table 5:** Hyperparameter values for rCCA Ridge and shrinkage methods.

Analysis performed	Recurrence site	Dataset	Penalisation method	Lambda 1 ($\lambda_{1}$)	Lambda 2 ($\lambda_{2}$)
rCCA	Local	Discovery	CV	0.2	0.11
			S	0.11	0.14
		Validation 1	CV	0.13	0.01
			S	0.17	0.36
		TCGA	CV	0.01	0.01
			S	0.15	0.14
	Liver	Discovery	CV	0.05	0.2
			S	0.07	0.09
		Validation 1	CV	0.02	0.02
			S	0.23	0.22
	Lung	Discovery	CV	0.03	0.05
			S	0.07	0.2
		Validation 1	CV	0.16	0.14
			S	0.15	0.32
	Peritoneal	Discovery	CV	0.07	0.18
			S	0.15	0.2
		Validation 1	CV	0.07	0.07
			S	0.29	0.49
	No recurrence	Discovery	CV	0.18	0.09
			S	0.07	0.13
		Validation 1	CV	0.02	0.18
			S	0.12	0.32
		GEO	CV	0.01	0.01
			S	0.11	0.12

Abbreviations: CV; Cross validation, S; Shrinkage.

**Table 6:** Upper and lower bounds of correlation values for rCCA for both gene expression and protein abundance sets.

Analysis performed	Penalisation method	Recurrence site	Upper bound		Lower bound
rCCA	Cross Validation (Ridge)	Liver	D	0.78	0.09
			V1	0.84	0.02
		Lung	D	0.64	0.06
			V1	0.6	0.03
		Peritoneal	D	0.5	0.04
			V1	0.71	0.04
		No recurrence	D	0.73	0.06
			V1	0.69	0.04
			V2	0.94	0.03
		Local	D	0.55	0.12
			V1	0.73	0.04
			V3	0.99	0.04
	Shrinkage	Liver	D	0.8	0.08
			V1	0.66	0.02
		Lung	D	0.77	0.09
			V1	0.74	0.05
		Peritoneal	D	0.71	0.09
			V1	0.8	0.05
		No recurrence	D	0.78	0.02
			V1	0.76	0.06
			V2	0.93	0.09
		Local	D	0.83	0.13
			V1	0.7	0.02
			V3	0.9	0.03

Abbreviations: rCCA; regularised canonical correlation analysis, SPLS; sparse partial least squares, D; Discovery cohort, V1; Validation 1 cohort, V2; Validation 2 cohort – GSE28735, V3; Validation cohort 3 – TCGA.

#### 4.4.2 Sparse partial least squares (SPLS)

We used SPLS [21,23] to associate proteomics data set with the transcriptomics data set. SPLS was chosen rather than traditional PLS [72], which maximises the covariance between the latent variables, and performs multi-omics integration with improved interpretability and feature selection [73]. SPLS applies LASSO or L1 penalisation to the loading vectors to execute feature selection [24]. To implement SPLS regression the first sparse PLS direction vector can be formulated as

$$\begin{array}{c} \max\left(\omega^{T}M\omega \right)\;subject\;to\;\omega^{T}\omega =1,\left|\omega \right|\leq \lambda \\ \;\omega \; \end{array}$$

Where $M=A^{T}BB^{T}A$, with A and B representing the gene expression and protein abundance data sets with the same samples but different features, respectively, and $A^{T}$ and $B^{T}$ denoting transformed versions of the matrices

and.

λ denotes the level of sparsity.

Using the first SPLS direction vector formulation detailed above, sparsity is applied using the L1 penalty onto an alternate direction vector (c) rather than the original direction vector (ω) to derive solution [74]

$$\min\left\{-\;\kappa \omega^{T}\omega +\left(1\;-\;\kappa \right)\left(c\;-\;\omega \right)+\lambda_{1}{\left|c\right|}_{1}+\lambda_{2}{\left|c\right|}_{2}^{2}\right\}\;subject\;to\;\omega^{T}\omega =1$$

Loading vectors for Y and X matrices were obtained when performing SPLS using *mixOmics* package [71] in R.

### 4.5 Script and data sets availability

The Rmarkdown files for all data analyses are accessible through figshare. A summary of the required packages used for all analyses are included in Table 7. Link: https://figshare.com/s/5e3df075c3b0664c73c9.

**Table 7:** Summary of packages employed in our study for performing relevant methods in the R statistical computing (R version 4.3.1) environment.

Method		Package name	Reference
Spearman partial correlation analysis		ppcor	[64]
Multivariate canonical correlation analysis	rCCA and SPLS	mixOmics	[71]
Data download and preparation		TCGAbiolinks GEOquery	[60,61]

Abbreviations: rCCA; regularised canonical correlation analysis, SPLS; sparse partial least squares.

## Funding

The authors acknowledge support from the 10.13039/501100000272NIHR Birmingham 10.13039/501100013629SRMRC, HYPERMARKER and the 10.13039/501100000265MRC Heath Data Research UK (HDRUK/CFC/01), an initiative funded by 10.13039/100014013UK Research and Innovation, Department of Health and Social Care (England) and the devolved administrations, and leading medical research charities. The views expressed in this publication are those of the authors and not necessarily those of the NHS, the National Institute for Health Research, the Medical Research Council or the Department of Health.

## Data availability

All data sets are publicly available.

## CRediT authorship contribution statement

**Animesh Acharjee:** Writing – review & editing, Writing – original draft, Supervision, Project administration, Methodology, Funding acquisition, Data curation, Conceptualization. **Daniella Okyere:** Writing – review & editing, Writing – original draft, Visualization, Methodology, Investigation, Formal analysis, Data curation. **Dipanwita Nath:** Writing – review & editing, Writing – original draft, Methodology, Formal analysis. **Shruti Nagar:** Writing – review & editing, Writing – original draft, Investigation, Formal analysis. **Georgios V. Gkoutos:** Writing – review & editing, Writing – original draft, Supervision, Funding acquisition, Conceptualization.

## Declaration of competing interest

The authors declare that they have no known competing financial interests or personal relationships that could have appeared to influence the work reported in this paper.
