# Supplementary material for: Network dynamics and therapeutic aspects of mRNA and protein markers with the recurrence sites of pancreatic cancer
Source: Heliyon. 2024 May 17;10(10):e31437. doi: 10.1016/j.heliyon.2024.e31437 (PMC11128524; doi:10.1016/j.heliyon.2024.e31437)
Supplement: Multimedia component 1 [file mmc1.docx]

# Supplementary Information

**Suppl. Table 1.1:** Spearman partial correlations in Discovery cohort.

| Discovery Cohort | | | | | |
| --- | --- | --- | --- | --- | --- |
| Liver networks | | | | | |
|  | Spearman partial correlation cutoff | | | | |
|  | r>0.75 | 0.75>r>0.65 | 0.65>r>0.55 | 0.55>r>0.45 | 0.45>r>0.35 |
| mRNA associations |  |  | ITGAM-PECAM1 (r=0.59091721) | HLA.DQ-CXCL10 (r=0.51171960) | CCL5-CXCL10 (r=0.37212394) |
|  | | | | | |
| Protein associations |  | STING-CD11c (r=0.6668589) |  | CD11c-FAP.alpha (r=0.4734789) | STING-CD66b (r=0.3792769) |
|  |  |  |  | SMA-FAP.alpha (r=0.5013983) | STING-FAP.alpha (r= - 0.3983113) |
|  |  |  |  |  | CD66b-FAP.alpha (r=0.3903316) |
|  | | | | | |
| Combined associations |  | STING-CD11c (r=0.65875672) | ITGAM-PECAM1 (r=0.57212522) | CD11c-FAP.alpha (r=0.52634162) | CCL5-CXCL10 (r=0.36566983) |
|  |  |  |  | SMA-FAP.alpha (r=0.46825061) | STING-CD66b (r=0.41010870) |
|  |  |  |  | HLA.DQ-CXCL10 (r=0.45718011) | STING-FAP.alpha (r= - 0.40808476) |
|  |  |  |  |  | CD66b-FAP.alpha (r=0.41217199) |
| Lung networks | | | | | |
|  | r>0.75 | 0.75>r>0.65 | 0.65>r>0.55 | 0.55>r>0.45 | 0.45>r>0.35 |
| mRNA associations |  |  | CCL5-PECAM1 (r=0.63032003) |  | HLA.DQ-CCL5 (r=0.36469629) |
|  | | | | | |
| Protein associations |  | STING-GZMB (r=0.66206305) |  |  | CD11c-GZMB (r=0.37876882) |
|  |  | SMA-FAP.alpha (r=0.70693910) |  |  |  |
|  | | | | | |
| Combined associations |  | STING-GZMB (r=0.670306834) | CCL5-PECAM1 (r=0.6488600) | ITGAM-STING (r=0.51662924) | CCL5-STING (r=0.37544702) |
|  |  |  | SMA-FAP.alpha (r=0.6249559460) | ITGAM-CD11c (r=0.48105580) | CD11c-GZMB (r=0.395300879) |
|  |  |  |  | STING-CD11c (r=-0.45334834) | LY6E-CXCL10 (r=0.35817495) |
| Local networks | | | | | |
|  | r>0.75 | 0.75>r>0.65 | 0.65>r>0.55 | 0.55>r>0.45 | 0.45>r>0.35 |
| mRNA associations | HLA.DQ-CCL5 (r=0.76548913) | ITGAM-PECAM1 (r=0.66401988) | ITGAM-CXCL10 (r=0.5789119) | CCL5-CXCL10 (r=0.5005437) | HLA.DQ-ITGAM (r=0.39096026) |
|  |  |  |  |  | HLA.DQ-CXCL10 (r= - 0.4115299) |
|  | | | | | |
| Protein associations |  | CD11c-FAP.alpha (r=0.6952068) | STING-CD66b (r=0.5698049) | STING-SMA (r=0.46794033) | STING-CD11c (r=0.4254414) |
|  |  | SMA-FAP.alpha (r=0.6952666) |  | STING-GZMB (r=0.46584905) | CD66b-FAP.alpha (r=0.3759208) |
|  | | | | | |
| Combined associations | HLA.DQ-CCL5 (r=0.824464176) | CD11c-FAP.alpha (r=0.696816196) | ITGAM-CXCL10 (r=0.55888341) | CCL5-CXCL10 (r=0.46659699) | HLA.DQ-ITGAM (r=0.37474578) |
|  |  | SMA-FAP.alpha (r=0.689463502) | STING-CD66b (r=0.60439849) | STING-SMA (r=0.487912335) | HLA.DQ-CXCL10 (r= - 0.39651006) |
|  |  |  | ITGAM-PECAM1 (r=0.63438020) | LY6E-CD66b (r=0.45693740) | STING-CD11c (r=0.40797340) |
|  |  |  |  |  | CXCL10-SMA (r= - 0.355299082) |
|  |  |  |  |  | STING-GZMB (r=0.44255840) |
|  |  |  |  |  | CD11c-SMA (r= - 0.361741788) |
| Peritoneal/other networks | | | | | |
|  | r>0.75 | 0.75>r>0.65 | 0.65>r>0.55 | 0.55>r>0.45 | 0.45>r>0.35 |
| mRNA associations |  |  | CCL5-CXCL10 (r=0.55276140) | ITGAM-PECAM1 (r=0.52359828) | LY6E-CCL5 (r=-0.38745419) |
|  |  |  |  |  | LY6E-PECAM1 (r=0.40562925) |
|  |  |  |  |  | CD44-CXCL10 (r=0.41410468) |
|  |  |  |  |  | CCL5-PECAM1 (r=0.43757602) |
|  | | | | | |
| Protein associations |  |  |  | CD11c-FAP.alpha (r=0.46887584) | STING-GZMB (r=0.37237314) |
|  |  |  |  |  | SMA-FAP.alpha (r=0.36296620) |
|  | | | | | |
| Combined associations |  |  | CCL5-CXCL10 (r=0.61940269) | LY6E-PECAM1 (r=0.482493002) | LY6E-CCL5 (r=-0.36655482) |
|  |  |  |  | ITGAM-PECAM1 (r=0.477127902) | CCL5-PECAM1 (r=0.410514315) |
|  |  |  |  |  | CD44-CXCL10 (r=0.39870833) |
|  |  |  |  |  | SMA-FAP.alpha (r=0.351856612) |
|  |  |  |  |  | HLA.DQ-CD66b (r=-0.355664385) |
|  |  |  |  |  | CCL5-GZMB (r=0.37524916) |
|  |  |  |  |  | CD44-SMA (r=0.39115551) |
|  |  |  |  |  | PECAM1-SMA (r=-0.36264123) |
|  |  |  |  |  | CD66b-GZMB (r=0.40383246) |
|  |  |  |  |  | CD11c-SMA (r=0.39339272) |
| No recurrence networks | | | | | |
|  | r>0.75 | 0.75>r>0.65 | 0.65>r>0.55 | 0.55>r>0.45 | 0.45>r>0.35 |
| mRNA associations |  |  |  |  | ITGAM-CCL5 (r=0.406161966) |
|  |  |  |  |  | ITGAM-PECAM1 (r=0.428183253) |
|  |  |  |  |  | CCL5-CXCL10 (r=0.395574190) |
|  | | | | | |
| Protein associations |  |  |  | STING-CD11c (r=0.48637969) | SMA-FAP.alpha (r=0.40979335) |
|  |  |  |  | CD11c-FAP.alpha (r=0.45557706) |  |
|  | | | | | |
| Combined associations |  |  |  | STING-CD11c (r=0.525777421) | ITGAM-CCL5 (r=0.40539019) |
|  |  |  |  | CD11c-FAP.alpha (r=0.47875807) | CCL5-CXCL10 (r=0.412519801) |
|  |  |  |  | ITGAM-PECAM1 (r=0.457793868) | CD44-STING (r=0.42230945) |
|  |  |  |  |  | SMA-GZMB (r=0.381824898) |
|  |  |  |  |  | CD66b-FAP-alpha (r=0.37484740) |

**Suppl. Table 1.2:** Spearman partial correlations in Validation- 1 cohort.

| Validation -1 Cohort | | | | | |
| --- | --- | --- | --- | --- | --- |
| Liver networks | | | | | |
|  | Spearman partial correlation cutoff | | | | |
|  | r>0.75 | 0.75>r>0.65 | 0.65>r>0.55 | 0.55>r>0.45 | 0.45>r>0.35 |
| mRNA associations |  | HLA.DQ-CXCL10 (r= 0.67997965) | CCL5-CXCL10 (r= 0.56058261) | HLA.DQ-ITGAM (r= 0.5381446) | LY6E-CXCL10 (r= 0.35553057) |
|  |  | ITGAM-PECAM1 (r= 0.705572521) |  | CCL5-PECAM1 (r=0.50246601) | LY6E-CCL5 (r= -0.37945419) |
|  |  |  |  | ITGAM-CXCL10 (r= -0.48602492) | CXCL10-PECAM1 (r= 0.403064431) |
|  | | | | | |
| Protein associations |  |  | STING-CD66b (r= 0.59261869) | STING-CD11c (r= 0.54161160) |  |
|  | | | | | |
| Combined associations |  | HLA.DQ-CXCL10 (r= 0.705318612) | HLA.DQ-ITGAM (r= 0.57895315) | ITGAM-CXCL10 (r= -0.512701215) | HLA.DQ-PECAM1 (r= -0.44049659) |
|  |  | ITGAM-PECAM1 (r= 0.70496261) | PECAM1-STING (r=0.579441616) | LY6E-CCL5 (r= -0.465548523) | STING-CD11c (r= 0.355921599) |
|  |  |  | STING-CD66b (r= 0.572483396) | CCL5-CXCL10 (r= 0.515116712) | CD44-FAP-alpha (r= 0.427213817) |
|  |  |  |  |  | ITGAM-CCL5 (r= 0.376866171) |
|  |  |  |  |  | LY6E-CD44 (r= -0.397229615) |
|  |  |  |  |  | CD44-GZMB (r= -0.38559397) |
|  |  |  |  |  | CXCL10-PECAM1 (r= 0.40289503) |
|  |  |  |  |  | SMA-FAP.alpha (r= 0.419631041) |
| Lung networks | | | | | |
|  | r>0.75 | 0.75>r>0.65 | 0.65>r>0.55 | 0.55>r>0.45 | 0.45>r>0.35 |
| mRNA associations |  | LY6E-CXCL10 (r=0.7268943) | CD44-PECAM1 (r=-0.5959773) | CXCL10-PECAM1 (r=0.4967931) | ITGAM-LY6E (r= -0.3624224) |
|  |  |  |  |  | LY6E-CD44 (r= -0.35445144) |
|  |  |  |  |  | CCL5-PECAM1 (r= 0.3695824) |
|  | | | | | |
| Protein associations |  |  | STING-GZMB (r= 0.568074135) |  | CD66b-SMA (r=0.4228827) |
|  |  |  | CD11c-GZMB (r= 0.589098665) |  |  |
|  | | | | | |
| Combined associations | CD11c-GZMB (r= 0.756242129) | CCL5-CD11c (r= 0.69736130) | CD44-PECAM1 (r= -0.64523404) | GZMB-SMA (r= 0.46694597) | CD44-STING (r= -0.39647944) |
|  |  |  | HLA.DQ-CCL5 (r= 0.55066528) | HLA.DQ-SMA (r= -0.46948255) | SMA-FAP.alpha (r= 0.408462391) |
|  |  |  | HLA.DQ-CD11c (r= -0.64813774) | ITGAM-CD66b (r= -0.50207008) | LY6E-PECAM1 (r= -0.37924607) |
|  |  |  | HLA.DQ-GZMB (r= 0.611013847) | CXCL10-PECAM1 (r= 0.48804170) | HLA.DQ-CD66b (r= 0.38919633) |
|  |  |  | LY6E-CXCL10 (r= 0.64240851) | STING-CD66b (r=0.51455377) | LY6E-CD44 (r= -0.40840151) |
|  |  |  | STING-GZMB (r= 0.553511652) |  | CCL5-GZMB (r= -0.421032414) |
|  |  |  | CD66b-SMA (r= 0.64438094) |  | CD44-CD66b (r= 0.42355614) |
|  |  |  |  |  | CXCL10-SMA (r= 0.35923502) |
|  |  |  |  |  | STING-CD11c (r= -0.37104366) |
|  |  |  |  |  | STING-SMA (r= -0.40085334) |
| Local networks | | | | | |
|  | r>0.75 | 0.75>r>0.65 | 0.65>r>0.55 | 0.55>r>0.45 | 0.45>r>0.35 |
| mRNA associations | ITGAM-CCL5 (r= 0.8340805) | ITGAM-PECAM1 (r= 0.69873568) |  | HLA.DQ-ITGAM (r= 0.4542671) | HLA.DQ-CXCL10 (r= -0.41243645) |
|  |  | ITGAM-LY6E (r= -0.7197979) |  |  | ITGAM-CD44 (r= 0.42113097) |
|  |  | LY6E-CCL5 (r= 0.7052421) |  |  | LY6E-CD44 (r= 0.38265470) |
|  |  | CD44-CXCL10 (r=-0.74704010) |  |  | LY6E-PECAM1 (r= 0.37582694) |
|  |  |  |  |  | CCL5-CD44 (r= -0.41109636) |
|  | | | | | |
| Protein associations |  |  | SMA-FAP.alpha (r= 0.61413520) |  | CD11c-GZMB (r= 0.36314941) |
|  |  |  |  |  | SMA-GZMB (r= -0.42983999) |
|  |  |  |  |  | FAP.alpha-GZMB (r= 0.41764224) |
|  |  |  |  |  | STING-GZMB (r= 0.41936148) |
|  | | | | | |
| Combined associations | ITGAM-LY6E (r= -0.80845108) | CD11c-FAP.alpha (r= -0.6579623) | STING-CD66b (r= 0.55278854) | STING-SMA (r= 0.5225127) | ITGAM-CD44 (r= -0.39111810) |
|  | ITGAM-PECAM1 (r= 0.862974167) | ITGAM-FAP-alpha (r= 0.6962523) | CCL5-STING (r=0.61148638) | CD66b-SMA(r= -0.5334046) | HLA.DQ-CXCL10 (r= -0.37706666) |
|  | CD44-FAP-alpha (r= 0.8654889) | LY6E-CCL5 (r= 0.667481749) | HLA.DQ-ITGAM (r= 0.602872953) | HLA.DQ-LY6E (r=0.45785912) | CXCL10-SMA (r= 0.3967742) |
|  | PECAM1-FAP-alpha (r= -0.9033233) | CCL5-CD66b (r= -0.67878288) | CD44-STING(r= 0.55145117) | HLA.DQ-PECAM1 (r= -0.451943301) | LY6E-FAP-alpha (r= 0.4190650) |
|  | CD66b-CD11c (r= 0.76605636) | CD44-PECAM1 (r= 0.741394871) | CD44-GZMB(r=-0.610804545) | ITGAM-CCL5 (r= 0.467495014) | CXCL10-FAP-alpha (r=-0.3646534) |
|  | SMA-FAP.alpha (r= 0.7953156) | CD44-CD66b (r= -0.74262640) | PECAM1-CD11c (r= -0.59614482) | LY6E-PECAM1 (r= 0.542134805) |  |
|  |  | CD44-CD11c (r= 0.72247670) | PECAM1-SMA(r=0.5604344) | CCL5-CD44 (r= -0.48285779) |  |
|  |  | CD44-SMA(r= -0.7254352) | STING-CD11c (r= -0.55852130) | CCL5-CD11c (r= 0.502227290) |  |
|  |  | STING-GZMB (r= 0.727002550) | CD66b-FAP-alpha(r=0.6070247) | CCL5-SMA (r= -0.4711082) |  |
|  |  | SMA-GZMB(r= -0.701848927) | CD11c-SMA (r= 0.5913710) | CCL5-GZMB (r= -0.484258359) |  |
|  |  |  | CD11C-GZMB(r= 0.575482587) | PECAM1-CD66b (r=0.50754936) |  |
|  |  |  |  | CD66b-GZMB(r= -0.5334046) |  |
|  |  |  |  | GZMB-FAP-alpha (r= 0.4882350) |  |
| Peritoneal/other networks | | | | | |
|  | r>0.75 | 0.75>r>0.65 | 0.65>r>0.55 | 0.55>r>0.45 | 0.45>r>0.35 |
| mRNA associations | HLA.DQ-CXCL10 (r= 0.7551337) | HLA.DQ-ITGAM (r= 0.7097591) | CD44-CXCL10 (r= 0.6433948) | CCL5-PECAM1 (r= - 0.5269948) | LY6E-CD44 (r= 0.3987027) |
|  | ITGAM-CXCL10 (r= -0.7517757) | ITGAM-CCL5 (r= 0.7171158) | HLA.DQ-CCL5 (r= -0.5826189) | CXCL10-PECAM1 (r= 0.4878149) | HLA.DQ-CD44 (r=-0.4292990) |
|  | ITGAM-PECAM1 (r= 0.7062535) |  | ITGAM-CD44 (r= 0.5618726) |  |  |
|  | CCL5-CXCL10 (r= 0.8849086) |  | LY6E-PECAM1 (r= 0.5926362) |  |  |
|  |  |  | CCL5-CD44 (r= -0.6406074) |  |  |
|  |  |  | CD44-PECAM1 (r= -0.5575271) |  |  |
|  | | | | | |
| Protein associations | GZMB-FAP-alpha (r= -0.8129458) | CD66b-FAP.alpha (r= 0.7260617) | CD66b-CD11c (r= 0.57700132) | CD11c-FAP.alpha (r= -0.5090987) | CD66b-SMA(r= 0.40537163) |
|  |  |  | CD66b-GZMB(r= 0.6420024) |  |  |
|  |  |  | CD11C-GZMB(r= -0.5752696) |  |  |
|  | | | | | |
| Combined associations | CCL5-CXCL10 (r= 0.86822019) | ITGAM-PECAM1 (r= 0.66293085) | HLA.DQ-ITGAM (r= 0.57927531) | ITGAM-CD66b (r=-0.54914022) | HLA.DQ-CCL5 (r= -0.4145382) |
|  | PECAM1-SMA (r= -0.85418566) | CD44-CXCL10 (r= 0.68625330) | HLA.DQ-STING (r= -0.64956667) | HLA.DQ-CXCL10 (r= 0.50882150) | HLA.DQ-GZMB (r= -0.36010603) |
|  | CD66b-CD11c (r= 0.8943813) | CD11c-CD44 (r= 0.6989392) | ITGAM-SMA (r= 0.63943552) | ITGAM-CD11c (r= 0.4583855) | HLA.DQ-CD66b (r= 0.41018643) |
|  | CD66b-GZMB(r= 0.94242407) | CD44-FAP-alpha (r= 0.68137426) | ITGAM-GZMB (r= 0.60780034) | LY6E-PECAM1 (r= 0.54605274) | CCL5-STING (r= -0.39780592) |
|  | CD66b-FAP-alpha (r= 0.76376758) | SMA-GZMB(r= -0.66907056) | LY6E-CD44 (r= 0.58170244) | LY6E-FAP.alpha (r= -0.52942969) | CXCL10-CD66b (r= 0.41306544) |
|  | CD11c-GZMB(r= -0.88416682) | CD66b-SMA(r= 0.67272180) | LY6E-STING (r=-0.57223572) | CD44-GZMB (r= 0.46465688) | CXCL10-GZMB (r= -0.36451051) |
|  | CD11c-FAP.alpha (r= -0.88869216) |  | PECAM1-CD66b (r= 0.55895076) | CXCL10-STING (r= 0.49572103) | CXCL10-FAP-alpha (r= -0.39766468) |
|  | GZMB-FAP-alpha (r= -0.81222348) |  | PECAM1-GZMB (r= -0.57172600) | CXCL10-CD11c (r= -0.5056654) | CD11c-SMA (r= -0.40596796) |
|  |  |  |  | CCL5-CD44 (r= -0.47482657) | LY6E-CD11c (r= -0.4386261) |
|  |  |  |  | CD44-CD66b (r= -0.48555888) | ITGAM-FAP-alpha (r= 0.44490860) |
|  |  |  |  |  | CCL5-CD66b (r= -0.38991484) |
| No recurrence networks | | | | | |
|  | r>0.75 | 0.75>r>0.65 | 0.65>r>0.55 | 0.55>r>0.45 | 0.45>r>0.35 |
| mRNA associations | ITGAM-CCL5 (r= 0.8653121) | CCL5-CXCL10 (r= 0.66059793) | HLA.DQ-CXCL10 (r= 0.63555747) | LY6E-CXCL10 (r= -0.46284459) | LY6E-PECAM1 (r= 0.4442352) |
|  | CXCL10-PECAM1 (r= 0.7763223) |  | HLA.DQ-PECAM1 (r=-0.6053962) |  | ITGAM-LY6E (r= -0.4443221) |
|  |  |  | ITGAM-CXCL10 (r= -0.62481965) |  | HLA.DQ-ITGAM (r= 0.3883618) |
|  |  |  | ITGAM-PECAM1 (r= 0.6325940) |  | HLA.DQ-LY6E (r=0.3610598) |
|  |  |  |  |  | LY6E-CCL5 (r= 0.3558937) |
|  |  |  |  |  | CCL5-PECAM1 (r= -0.4025695) |
|  | | | | | |
| Protein associations |  |  | CD11c-SMA (r= 0.59641202) | STING-FAP-alpha (r= 0.48759862) | SMA-FAP.alpha (r= 0.38222576) |
|  |  |  |  |  | STING-SMA(r= -0.37370061) |
|  |  |  |  |  | GZMB-FAP-alpha (r= 0.44859635) |
|  | | | | | |
| Combined associations | ITGAM-CCL5 (r= 0.8074379) | CCL5-CXCL10 (r= 0.73960965) | ITGAM-CXCL10 (r= -0.57628989) | HLA.DQ-LY6E (r= 0.479256459) | HLA.DQ-ITGAM (r= 0.4166301) |
|  | ITGAM-PECAM1 (r= 0.77386538) | CXCL10-PECAM1 (r= 0.71689281) | LY6E-PECAM1 (r= 0.55002913) | HLA.DQ-CXCL10 (r= 0.50586124) | HLA.DQ-SMA (r= 0.43653140) |
|  |  | STING-FAP-alpha (r= 0.678026609) | LY6E-CD11c (r= 0.61248248) | HLA.DQ-PECAM1 (r= -0.54674705) | ITGAM-STING (r= 0.370302674) |
|  |  |  | CD11c-SMA (r= 0.60785988) | ITGAM-LY6E (r= -0.511827514) | LY6E-CXCL10 (r= -0.37099013) |
|  |  |  |  | CCL5-PECAM1 (r= -0.54347410) | LY6E-SMA (r= -0.44642206) |
|  |  |  |  | CCL5-CD66b (r= -0.531544124) | CCL5-CD44 (r= -0.38857411) |
|  |  |  |  | CXCL10-CD66b (r= 0.484647773) | CCL5-GZMB (r= 0.41975827) |
|  |  |  |  | CD66b-GZMB (r=0.53773020) | CXCL10-GZMB (r= -0.39847284) |
|  |  |  |  |  | PECAM1-CD11c (r= -0.41768678) |
|  |  |  |  |  | CD66b-SMA(r= -0.38959200) |

**Suppl. Table 1.3:** Spearman partial correlations in Validation- 2 and Validation- 3 cohorts.

| Validation -2 Cohort; GEO GSE28735 | | | | | |
| --- | --- | --- | --- | --- | --- |
| Local networks | | | | | |
|  | Spearman partial correlation cutoff | | | | |
|  | r>0.75 | 0.75>r>0.65 | 0.65>r>0.55 | 0.55>r>0.45 | 0.45>r>0.35 |
| mRNA associations |  |  | ITGAM-PECAM1 (r= 0.55959) | LY6E-CCL5 (r= 0.47574247) |  |
|  |  |  |  | CD44-CCL5 (r= -0.49684664) |  |
|  | | | | | |
| Protein associations |  |  | STING-SMA (r= 0.64843461) |  | STING-CD11c (r= 0.37898972) |
|  |  |  | SMA-FAP.alpha (r= 0.61900042) |  |  |
|  | | | | | |
| Combined associations |  |  | LY6E-STING (r= 0.55721605) | LY6E-FAP.alpha (r= 0.51067789) | CD44-CCL5 (r= -0.44093745) |
|  |  |  | ITGAM-CD11c (r= 0.634232905) | STING-FAP.alpha (r= -0.46438124) | CD44-CD11c (r= 0.435389492) |
|  |  |  |  | ITGAM-STING (r= 0.47365492) | CXCL10-FAP.alpha (r= 0.37568367) |
|  |  |  |  | HLA.DQ-CXCL10 (r= 0.49345639) | PECAM1-FAP.alpha (r= 0.38815658) |
|  |  |  |  | CXCL10-PECAM1 (r= -0.50004489) | LY6E-CD66b (r= 0.40332092) |
|  |  |  |  | CXCL10-SMA (r= 0.53903353) | HLA.DQ-CD11c (r= -0.36915679) |
|  |  |  |  |  | PECAM1-STING (r= 0.41954228) |
|  |  |  |  |  | PECAM1-SMA (r= 0.36815065) |
| Validation -3 Cohort; TCGA PAAD | | | | | |
| No recurrence networks | | | | | |
|  | r>0.75 | 0.75>r>0.65 | 0.65>r>0.55 | 0.55>r>0.45 | 0.45>r>0.35 |
| mRNA associations |  |  |  |  | HLA.DQ-ITGAM (r= 0.36442049) |
|  |  |  |  |  | HLA.DQ-CCL5 (r= 0.38569325) |
|  |  |  |  |  | LY6E-PECAM1 (r= -0.36480541) |
|  |  |  |  |  | CCL5-CXCL10 (r= 0.35791268) |
|  |  |  |  |  | CCL5-PECAM1 (r= 0.36272637) |
|  | | | | | |
| Protein associations |  | SMA-FAP.alpha (r= 0.69948902) |  |  |  |
|  | | | | | |
| Combined associations |  | SMA-FAP.alpha (r= 0.660202281) |  | CD11c-ITGAM (r= 0.502212296) | CCL5-GZMB (r= 0.43825853) |
|  |  |  |  | STING-LY6E (r= 0.511987526) | CD66b-ITGAM(r= 0.44082866) |
|  |  |  |  |  | LY6E-PECAM1 (r= -0.37606051) |

**Suppl. Table 2:** Reported gene targets of the mRNA analysis

| Target name | Target group membership(s) |
| --- | --- |
| CD40 | Tumor Necrosis Factor Receptor Superfamily Member 5 |
| LAG3 | T cells, Checkpoint, T cell Activation |
| CCND1 | Proliferation |
| IFNGR1 | Interferon |
| IL6 | Cytokine & Chemokine Signaling |
| IL15 | Cytokine & Chemokine Signaling |
| PDCD1 | T cells, Checkpoint, T cell Activation |
| PDCD1LG2 | Checkpoint |
| CD4 | T cells, Th cells |
| BCL2 | Tumor, Apoptosis |
| KRT | Epithelial, Tumor |
| IFNG | Interferon |
| ARG1 | Myeloid Activation, Myeloid Suppression, Myeloid, M2 Macrophage, Macrophage |
| PSMB10 | Antigen Presentation |
| AKT1 | Tumor |
| HLA-DQ | MHC2, Antigen Presentation |
| STAT3 | Cytokine & Chemokine Signaling |
| CD8A | T cells, CD8 T cells |
| CD3E | T cells |
| ICAM1 | Immune Cell Adhesion & Migration |
| HLA-DRB | MHC2, Antigen Presentation |
| CTLA4 | T cells, Checkpoint, T cell Activation, Th cells |
| CD74 | MHC2, Antigen Presentation |
| IDO1 | Myeloid Suppression, Myeloid |
| CD276 | Checkpoint |
| LY6E | T cells, Myeloid |
| PTPRC | Total Immune |
| IL12B | Cytokine & Chemokine Signaling |
| B2M | Tumor, Antigen Presentation |
| CTNNB1 | Tumor, Wnt Signaling |
| ITGAV | Immune Cell Adhesion & Migration |
| IFNAR1 | Interferon |
| STAT1 | Cytokine & Chemokine Signaling |
| FOXP3 | T cells, Tregs, Th cells |
| TNFRSF9 | T cells, T cell Activation |
| CCL5 | Cytokine & Chemokine Signaling |
| CXCL10 | Cytokine & Chemokine Signaling |
| ITGAM | Immune Cell Adhesion & Migration |
| NKG7 | Cytotoxicity |
| ITGB8 | Immune Cell Adhesion & Migration |
| FAS | Apoptosis, Cytokine & Chemokine Signaling |
| BATF3 | Dendritic cells |
| CD274 | Myeloid Activation, Checkpoint |
| CD44 | T cell Activation |
| TIGIT | T cells, Checkpoint |
| TBX21 | Th cells |
| VSIR | Myeloid Activation, Checkpoint, Myeloid, Macrophage |
| CD27 | T cells, T cell Activation |
| CMKLR1 | Cytokine & Chemokine Signaling |
| CXCL9 | Cytokine & Chemokine Signaling |
| HIF1A | Tumor, Cytokine & Chemokine Signaling |
| ICOSLG | Checkpoint, Myeloid, B cells |
| DKK2 | Wnt Signaling |
| TNF | Apoptosis, Cytokine & Chemokine Signaling |
| CD47 | Myeloid |
| GZMB | T cell Activation, Cytotoxicity |
| MS4A1 | B cells |
| CSF1R | Myeloid Activation, Myeloid, Cytokine & Chemokine Signaling |
| CD86 | Checkpoint, T cell Activation, Myeloid |
| CXCR6 | Cytokine & Chemokine Signaling |
| STAT2 | Cytokine & Chemokine Signaling |
| HLA-E | Antigen Presentation |
| PECAM1 | Immune Cell Adhesion & Migration |
| ITGB2 | Immune Cell Adhesion & Migration |
| HAVCR2 | T cells, Checkpoint, T cell Activation |
| EPCAM | Epithelial, Tumor |
| CD40LG | T cells |
| PTEN | Tumor |
| CD68 | Macrophage |
| ITGAX | Immune Cell Adhesion & Migration |
| VEGFA | Cytokine & Chemokine Signaling |
| MKI67 | Proliferation |

**Suppl. Table 3:** Reported protein targets of the Immune Cell Profiling Core

| Protein name | Gene | Full target name |
| --- | --- | --- |
| CD45 | PTPRC | protein tyrosine phosphatase, receptor type, C |
| CD3 | CD3G, CD3E, CD3D | CD3g molecule, gamma (CD3-TCR complex), CD3e molecule, epsilon (CD3-TCR complex), CD3d molecule, delta (CD3-TCR complex) |
| CD4 | CD4 | CD4 molecule |
| CD8 | CD8A | CD8a molecule |
| CD20 | MS4A1 | membrane-spanning 4-domains, subfamily A, member 1 |
| CD11c | ITGAX | integrin, alpha X (complement component 3 receptor 4 subunit) |
| CD68 | CD68 | CD68 molecule |
| CD163 | CD163 | CD163 molecule |
| FOXP3 | FOXP3 | forkhead box P3 |
| CD66b | CEACAM8 | carcinoembryonic antigen-related cell adhesion molecule 8 |
| CD45RO | PTPRC | protein tyrosine phosphatase, receptor type, C |
| CD14 | CD14 | CD14 molecule |
| CD25 | IL2RA | interleukin 2 receptor, alpha |
| CD27 | CD27 | CD27 molecule |
| CD34 | CD34 | CD34 molecule |
| CD40 | CD40 | CD40 molecule, TNF receptor superfamily member 5 |
| CD44 | CD44 | CD44 molecule (Indian blood group) |
| CD56 | NCAM1 | neural cell adhesion molecule 1 |
| CD80 | CD80 | CD80 molecule |
| CD127 | IL7R | interleukin 7 receptor |
| PD-1 | PDCD1 | programmed cell death 1 |
| PD-L1 | CD274 | CD274 molecule |
| PD-L2 | PDCD1LG2 | programmed cell death 1 ligand 2 |
| HLA-DR | CD74 | CD74 molecule, major histocompatibility complex, class II invariant chain |
| GZMB | GZMB | granzyme B |
| CTLA4 | CTLA4 | cytotoxic T-lymphocyte associated protein 4 |
| STING | TMEM173 | transmembrane protein 173 |
| IDO1 | IDO1 | indoleamine 2,3-dioxygenase 1 |
| Tim3 | HAVCR2 | hepatitis A virus cellular receptor 2 |
| 4-1BB | TNFRSF9 | tumor necrosis factor receptor superfamily, member 9 |
| OX40L | TNFSF4 | tumor necrosis factor superfamily member 4 |
| LAG3 | LAG3 | lymphocyte-activation gene 3 |
| VISTA | **VSIR** | V-set immunoregulatory receptor |
| ICOS | ICOS | inducible T-cell co-stimulator |
| GITR | TNFRSF18 | TNF receptor superfamily member 18 |
| ARG1 | ARG1 | arginase 1 |
| Beta2 Microgl | B2M | beta-2-microglobulin |
| B7-H3 | CD276 | CD276 molecule |
| Ki-67 | MKI67 | marker of proliferation Ki-67 |
| SMA | ACTA2 | actin, alpha 2, smooth muscle, aorta |
| Fibronectin | FN1 | fibronectin 1 |
| FAP-alpha | FAP | fibroblast activation protein alpha |

**Supplementary Table 4**. Comparison of clinical and pathological variables between High, Intermediate, and Low grade budding patients for Validation 1 dataset (n = 109). Quantitative data are presented as group medians and interquartile range (IQR) values are specified in brackets.

| **Characteristic** | **High grade** | **Intermediate grade** | **Low grade** | **p-value** |
| --- | --- | --- | --- | --- |
| Number (%) | 40 (36.7) | 40 (36.7) | 29 (26.6) |  |
| OS (months) | 11 (8 – 20) | 16 (10 – 28.3) | 20 (15 – 75) | 1.149e-13 |
| DFS (months) | 6 (4 – 12.5) | 8.5 (6 – 24) | 16 (9 – 68) | 3.801e-10 |
| Sex, n (%) | | | | 0.112 |
| F | 16 (40) | 20 (50) | 19 (65.5) |  |
| M | 24 (60) | 20 (50) | 10 (34.5) |  |
| Age | 67 (55 – 75) | 65.5 (61.8 – 71) | 63 (54 – 75) | < 2.2e-16 |
| CA19-9 (U/mL) | 540 (58 – 1500) | 118.5 (0 – 296) | 145 (52 – 380) | 5.054e-11 |
| Size (mm) | 35.5 (30 – 51.3) | 32.5 (20 – 40) | 30 (25 – 35) | < 2.2e-16 |
| Buds Number x20 | 28.5 (8.8 – 42) | 11 (7.8 – 22.3) | 7 (2 – 17) | < 2.2e-16 |
| Budding ITBCC | 23.5 (7.2 – 34.7) | 8.7 (5.8 – 18.8) | 5.8 (1.7 – 14.0) | < 2.2e-16 |
| Budding category, n (%) | | | | <0.001 |
| 1 | 4 (10) | 5 (12.5) | 14 (48.3) |  |
| 2 | 8 (20) | 16 (40) | 7 (24.1) |  |
| 3 | 28 (70) | 19 (47.5) | 8 (27.6) |  |
| Organ, n (%) | | | | 0.074 |
| Liver | 13 (32.5) | 13 (32.5) | 7 (24.1) |  |
| Local | 4 (10) | 6 (15) | 4 (13.8) |  |
| Lung | 9 (22.5) | 7 (17.5) | 4 (13.8) |  |
| Other | 2 (6.9) | 3 (7.5) | 10 (25) |  |
| No recurrence | 12 (41.4) | 11 (27.5) | 4 (10) |  |
